# Low utility of Oncotype DX® in the clinic

**DOI:** 10.1002/cam4.837

**Published:** 2017-02-01

**Authors:** Luisel J. Ricks‐Santi, John Tyson McDonald

**Affiliations:** ^1^ Cancer Research CenterHampton University100 E. Queen StreetHamptonVirginia23668

**Keywords:** Breast cancer, genomics, health disparities, oncotypeDX, precision medicine

## Abstract

Precision medicine tools are currently making their way into the clinic and being utilized to diagnose, prognose, and individualize cancer care. The multi‐gene expression‐based assay, Oncotype DX® (ODX), is a genomic tumor profiling tool that determines the expression of 21 tumor‐ associated genes; it helps determine the risk for distant recurrence and whether chemotherapy is an appropriate course of treatment in patients with early stage, estrogen receptor (ER) positive, HER2 negative, and lymph node negative (or 1–3 positive lymph nodes) invasive BCa. The aim of this study was to determine the overall utilization and uptake of the ODX genomic test in a cross‐sectional analysis of the Virginia Tumor registry, compare utilization in African Americans (AAs) and Caucasian Americans (CAs), and determine the profile of patients referred for testing. Caucasian (89.7%) patients made up the majority of the ODX testers compared to AAs (10.3%) (*P* < 0.0001). Those who received ODX testing were less likely to have higher grade and higher stage tumors, and were less likely to be ER negative (RR = 0.21, 95% CI: 0.01–0.31), progesterone receptor (PR) negative (RR = 0.35, 95% CI: 0.27–0.45), HER2 amplified (RR = 0.27, 95% CI: 0.17–0.43), or triple negative (RR = 0.21, 95% CI: 0.14–0.33). Of the patients that were eligible (*n* = 3924), 10.5% (*n* = 412) received ODX testing. Specifically, 11.7% of the Caucasian patients and 5.1% of AAs patients received ODX testing (*P* < 0.001). Our analysis confirmed that the utilization of ODX was low and that AAs were much less likely to receive ODX testing.

## Introduction

It is well known that breast cancer (BCa) is a heterogeneous disease composed of several histologies and “intrinsic” molecular subtypes identified by microarray analysis which appear to be associated with prognosis 1, 2, 3, 4, 5, 6, 7. The intrinsic molecular BCa subtypes correlate with the presence or absence of three hormone receptors, estrogen receptor (ER), progesterone receptor (PR), and human epidermal growth factor receptor 2 (HER2), which are highly indicative of prognosis and also informs successful treatment options 8. These receptors are targets for BCa treatment and prevention: Tamoxifen targets the estrogen receptor (ER), while herceptin targets the human epidermal growth factor receptor 2 (HER2); without expression of these hormone receptors, treatment is limited to toxic chemotherapeutic options.

Another innovative way to determine the best course of treatment for BCa is to use genomic tools which recently have fundamentally changed the way oncologists treat cancer 9. The multi‐gene expression‐based assay, Oncotype DX (ODX‐Genomic Health, Redwood City, CA), is a genomic tumor profiling tool that determines the expression of 21 tumor‐associated genes; it helps determine the risk for distant recurrence and whether chemotherapy is an appropriate course of treatment in patients with early stage, ER positive, HER2 negative, and lymph node negative (or 1–3 positive lymph nodes) invasive BCa. Indeed, while the use of adjuvant systemic chemotherapy mostly benefits those who cannot be cured by surgery or radiation alone, in those patients with localized disease, there is little benefit and adverse effects are substantial. Therefore, in line with other breast cancer treatment modalities, the application of chemotherapy is becoming increasingly individualized, while attempting to avoid overtreatment.

Although BCa is one of the most common malignancies in women, the measure of the clinicopathological features necessary to be eligible for ODX testing such as ER receptor status and early stage tumors, are disproportionately distributed among populations; as such, the intrinsic molecular subtypes, which overlap the hormone receptor status, are also disproportionately distributed among populations. Luminal A tumors, characterized by the strong expression of ER or PR and HER2 negativity are more prevalent in women of European descent, while the triple‐negative subtype, which lacks the expression of ER, PR, and HER2, has a much higher prevalence in women of African descent 10. We surmise that because of the high prevalence of ER negative disease in women of African descent, the usefulness of ODX remains restricted in the population resulting in the limited availability of genomic tools for patients with increasingly adverse BCa prognoses. Therefore, the aim of this study was to determine the overall utilization of the ODX genomic test in a cross‐sectional analysis of the Virginia Tumor registry, compare utilization in African Americans (AAs) and Caucasian Americans (CAs), and determine the profile of patients referred for testing.

## Methods

### Data source and study sample

This study utilized data from the Virginia Tumor Registry. Eligibility criteria for this study included all histologically confirmed malignant BCas in Virginia, diagnosed between 2000 and 2012. Patients with duplicate records and multiple diagnoses were consolidated and coded as recurrences leaving 62,838 women with BCa diagnoses. All benign and stage 0 cases were excluded, as well as patients of ethnicities other than AAs and CAs. Patients with missing data for the following variables: age at diagnosis, stage, grade, tumor size, ER status, and PR status were excluded leaving 9,120 patient records available for analysis. Self‐identified patient race was measured in two categories representing CAs and AAs. Total ODX utilization was measured as a function of those eligible who utilized ODX divided by total eligible in a cross‐sectional analysis of cases between 2009 and 2012. Additionally, ODX utilization was stratified by race.

### Study variables

Demographic (race and age) and clinicopathological characteristics were first compared in all CAs versus AAs. Specifically, age at diagnosis, tumor size, stage at diagnosis, grade at diagnosis, estrogen receptor status, progesterone receptor status, HER2 status, and molecular subtype were compared. ER and PR status was determined by immunohistochemistry (IHC) and HER2 was determined by fluorescence in situ hybridization (FISH) or IHC. Molecular subtype was indicated by hormone receptor ER and PR status and HER2/neu status. These statuses were analyzed independently or together to determine the molecular subtype. For tumors with ER, PR, and HER2 data, tumors that were ER and/or PR positive and HER2 negative were coded as luminal A; tumors that were ER and/or PR positive and HER2 positive were coded as luminal B; tumors that were ER and/or PR negative and HER2 positive were coded as HER2 overexpressing/amplified; and tumors negative for ER, PR, and HER2 were coded as triple negative. Tumors were also characterized for ER and PR only, if HER2 status was not available. Stage was measured in four categories (stage 1, stage 2, stage 3, and stage 4). Grade was measured in terms of three categories: low I), intermediate (II), and high (III). The ODX eligibility criteria (early stage, ER positive, HER2 negative, node negative, node positive [1‐2]) was used to select all patients eligible for ODX testing. The first case where ODX was utilized was in 2009. Therefore, only cases between 2009 and 2012 were included in the cross‐sectional analysis.

### Statistical analysis

Frequency distributions, chi‐square tests, and Fisher exact tests were used to compare racial differences by clinicopathological characteristics and to compare the differences among the patients (eligible and ineligible) who received ODX testing compared to those who did not. To determine the association between receiving ODX testing, race, and clinicopathological variables (i.e., ER status, PR, status, grade, and stage), the risk ratio was calculated using binary logistic regression. Differences between CA and AA ODX users were also measured using chi‐square test. *P*‐values less than 0.05 were considered statistically significant.

## Results

**Figure 1:**
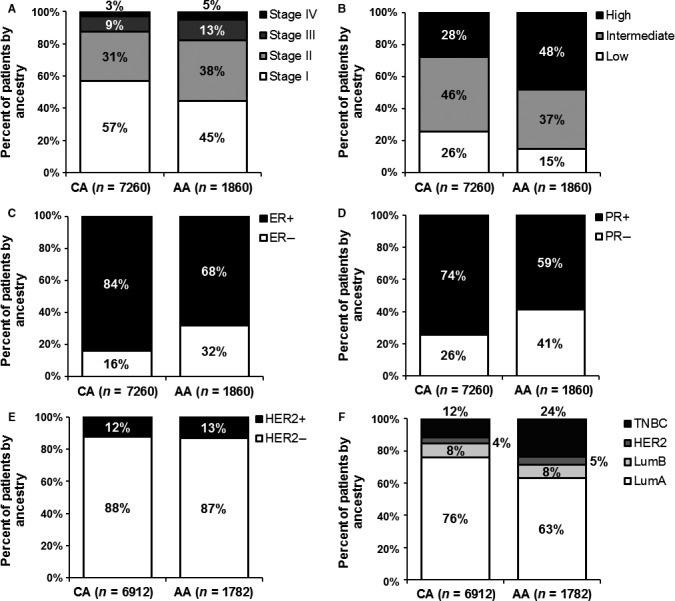
Breast cancer clinicopathological differences comparing Caucasian American and African American cases, 2000–2012.

In general, AAs breast cancer patients were more likely to be younger (mean=58.99 ± 13.30 years) and have larger tumors (26.93 ± 35.10 mm) compared to Caucasians (mean age= 62.43 ± 17.25 years; 20.55 ± 23.52). The clinicopathological differences between CA and AA cases can be found in figure 1. There was a statistically significant trend for AAs present with later stage (*P* < 0.001) and higher grade tumors (*P* < 0.001). AAs compared to CAs had a significantly higher frequency of ER negative (*P* = <0.001), PR negative (*P* < 0.001), triple negative, and double negative (28.8% vs. 15.5%; *P* < 0.001 [data not shown]) breast cancer (Figure 1).

A cross‐sectional analysis was performed on patients who had undergone ODX testing between 2009 (when the first test was administered) and 2012. Caucasian (89.7%) patients made up the majority of the ODX testers compared to AAs (10.3%) (*P* < 0.0001) (Table 1). Those who received ODX testing were less likely to have higher grade and higher stage tumors, and were less likely to be ER negative (RR = 0.21, 95% CI: 0.01–0.31), PR negative (RR = 0.35, 95% CI: 0.27–0.45), HER2 amplified (RR = 0.27, 95% CI: 0.17–0.43), or triple negative (RR = 0.21, 95% CI: 0.14–0.33).

**Table 1:** Association of demographic and clinicopathological variables with performance of Oncotype DX

		No	%	Yes	%	Risk ratio	95% CI lower	95% CI upper	*P*‐value
		*n* = 5182		*n* = 446					
Race									
Caucasian		3964	76.5%	400	89.7%				
African American		1218	23.5%	46	10.3%	0.402	0.2994	0.5407	<0.001
Grade 2									
I		1126	21.7%	141	31.6%				
II		2177	42.0%	226	50.7%	0.8451	0.6926	1.0312	0.11
III		1879	36.3%	79	17.7%	0.3626	0.2524	0.4466	<0.001
Stage									
Stage 1		2598	50.1%	296	66.4%				
Stage 2		1677	32.4%	129	28.9%	0.5989	0.4908	0.7308	<0.001
Stage 3		665	12.8%	18	4.0%	0.2577	0.1613	0.4116	<0.001
Stage 4		242	4.7%	1	1.2%	0.0402	0.0057	0.2853	<0.001
Estrogen receptor status									
Positive		3904	75.3%	419	93.9%				
Negative		1278	24.7%	27	6.1%	0.21	0.01454	0.3135	<0.001
Progesterone receptor status									
Positive		3401	65.6%	381	85.4%				
Negative		1781	34.4%	65	14.6%	0.3495	0.2703	0.452	<0.001
HER2 status									
Not amplified		4208	85.1%	413	95.8%				
Amplified		736	14.9%	18	4.2%	0.2671	0.1677	0.4255	<0.001
Hormone receptor status									
ER and PR positive		3319	64.0%	381	85.4%				
ER or PR positive		667	12.9%	38	8.5%	0.2609	0.1543	0.441	<0.001
Double negative		1196	23.1%	27	6.1%	0.209	0.1423	0.307	<0.001
Subtype (Only in patients with ER, PR, and HER2 data)									
Luminal A		3303	66.8%	392	91.0%				
Luminal B		494	10.0%	14	3.2%	0.2598	0.1537	0.4391	<0.001
HER2 overexpressing		242	4.9%	4	0.9%	0.1533	0.0577	0.4069	<0.001
Triple negative		905	18.3%	21	4.9%	0.2138	0.1386	0.3296	<0.001

ER, estrogen receptor; PR, progesterone receptor.

Of the patients who were eligible (*n* = 3924), 10.5% (*n* = 412) received ODX testing. Specifically, 11.7% of the Caucasian patients and 5.1% of AAs patients received ODX testing (*P* < 0.001) (Table 2). It is also notable that between 2009 and 2011, ODX testing in eligible patients increased from 4.7% in 2009 to 9.5% in 2012 (data not shown); however, when stratified by race, ODX testing in eligible AAs only increased from 1.5% in 2009 to only 5.1% in 2012 (data not shown).

**Table 2:** Differences in utility of Oncotype DX between Caucasians and AAs breast cancer cases between 2009 and 2012

	Caucasian	%	African American	%	*P*‐value
Eligible for Oncotype DX and results could be assessed	5677		1141		
Oncotype DX performed?					
No	2841	88.3%	689	94.9%	
Yes	375	11.7%	37	5.1%	0.000
Unknown or could not be assessed	2461	43.40%	415	42.20%	

	Caucasian			African American		
	Eligible	Performed (*n* = 375)		Eligible	Performed (*n* = 37)	
2009	4	0	0.0%	2	0	0.0%
2010	2702	137	5.1%	536	8	1.5%
2011	2971	238	8.0%	603	29	4.8%

An analysis of the characteristics of the patients receiving ODX testing was also performed. AAs were younger (*P* = 0.014), had larger tumors (*P* = 0.066), had a lower frequency of stage 1 and 2 tumors, and a higher frequency of higher grade (*P* = 0.002), ER negative (*P* = 0.003), double negative (*P* = 0.002) and triple negative (*P* = 0.02) tumors (Table 3). ODX scores were not significantly different between CAs and AAs, although there was a trend for higher scoring tumors in AAs.

**Table 3:** Clinicopathological characteristics in Caucasian Americans and AA Oncotype DX users

		Caucasians	%	African American	%	*P*‐value
		*n* = 400		*n* = 46		
Mean age		57.84 (±10.912)		53.85 (±11.849)		0.014
Mean tumor size		28.92 (±104.686)		60.38 (±188.742)		0.066
Stage						
1		272	68.0%	24	52.2%	
2		110	27.5%	19	41.3%	
3		17	4.3%	1	2.2%	
4		1	0.3%	2	4.3%	0.002
Grade						
I		125	31.3%	16	34.8%	
II		212	53.0%	14	30.4%	
III		63	15.8%	16	34.8%	0.002
ER						
Positive		381	95.3%	38	82.6%	
Negative		19	4.8%	8	17.4%	0.003
PR						
Positive		345	86.3%	36	78.3%	
Negative		55	13.8%	10	21.7%	0.112
HER2						
Not amplified		369	95.6%	44	97.8%	
Amplified		17	4.4%	1	2.2%	0.421
Molecular subtype						
Luminal A		356	92.2%	36	80.0%	
Luminal B		13	3.4%	1	2.2%	
HER2 overexpressing		4	1.0%	0	0.0%	
Triple negative		13	3.4%	8	17.8%	0.02
Oncotype DX score						
Low risk (good prognosis)		199	73.4%	20	58.8%	
Intermediate risk of recurrence		31	11.4%	5	14.7%	
High risk (poor prognosis)		41	15.1%	9	26.5%	0.17
Subtype 2						
ER and PR positive		345	86.30%	36	78.3%	
ER or PR positive		36	9.00%	2	4%	
ER and PR negative		19	4.80%	8	17.4%	0.002

ER, estrogen receptor; PR, progesterone receptor.

## Conclusions

Consistent with several BCa epidemiological studies 10, 11, 12, 13, 14, 15, ER negative and triple negative BCa (TNBC) displayed disproportionate frequencies in AA women in VA and were therefore less likely to be eligible for ODX testing. Furthermore, AAs presented with larger, higher stage and higher grade tumors, as well as being younger at diagnosis. Our analysis demonstrated that in Virginia, the utilization of ODX was much lower than other published studies (10–35%) and that AAs were much less likely to receive ODX testing 16, 17, 18, 19, 20; there was also a trend for higher scores in AAs, although not statistically significant. Our results also confirmed that patients with ER negative, double negative, and triple negative breast cancer, were also less likely to receive ODX testing consistent with the indications for utilization of ODX.

Precision medicine tools are currently making their way into the clinic and being utilized to diagnose, prognose, and individualize cancer care 21, 22, 23, 24. The ODX test is used to determine which early stage tumors may benefit from adjuvant chemotherapy. Eligible patients include those with ER‐positive disease who may benefit from endocrine therapy, as well as chemotherapy to reduce the risk for recurrence resulting in increased survival and improved quality of life. Thanks to advances in genomic testing and deeper insights into the heterogeneity of breast cancer, the physicians are learning that the one‐size‐fits‐all approach is not effective in treating breast cancer. Furthermore, research now shows that for some women with stages 1 and 2 breast cancer, the absolute survival benefit from preventive double mastectomies is less than 1% after 20 years and that some women with early‐stage breast cancer do not benefit from chemotherapy. Genomic testing could reduce the number of patients subjected to surgery, radiation, and/or chemotherapy unnecessarily. Quality of life is also becoming more and more important as survivorship increases. While 5‐year survival rates are different between AAs and CAs, local BCa survival rates between the groups are virtually equal (94% and 99%, respectively). The use of ODX could not only help improve survivorship but could also lend itself to the improvement of quality of life as some patients may avert the use of toxic chemotherapy if their risk for recurrence is low.

Consistent with a study by Lund et al. 16, our study also found a trend for higher recurrence scores in AAs which correlates with increased mortality. Lund et al. suggests that these differences could indicate that tumors in AAs are more likely to have decreased ER expression, potentially resulting in endocrine therapy unresponsiveness, and thus increased adverse outcomes. Still, while ODX presents a unique opportunity to individualize treatment and improve outcomes in BCa patients, AA women are less likely to benefit because of the adverse clinicopathological characteristics presented by their tumors. Patients of African descent are disproportionately affected with ER negative and later stage disease making many of these patients and others with ER‐negative disease ineligible for these life‐saving genomic tools.

When AAs meet the eligibility criteria, they are still less likely to be tested by ODX, consistent with several studies done in Atlanta 25, New York City 18, North Carolina 20, and nationally 17, 19. We also show a lower than reported use of ODX in our population. This could be a reflection of the few academic cancer centers in Virginia; physicians at academic cancer centers have been suggested to engage more readily in ODX's usage 19. Others have cited that ODX was more likely to be performed at private hospitals compared to inner‐city and municipal hospitals 16, 18. Moreover, Dinan et al. found that patients living in rural areas were less likely to receive ODX testing 19. These data suggest that physicians serving underserved populations may lack the confidence or understanding necessary to make referrals for ODX limiting their use in minority and underserved populations, although covered by insurance and Medicare. Consistent with this finding, a study aiming to determine the decision‐making styles of patients reported that the most common reason women eligible for ODX did not receive it was because their doctor did not offer it to them (80%) or that they had not heard of it (65%) 26. The impact of the disproportionate use of genomic tools for cancer treatment decision making is far reaching and could consequently increase or allow cancer disparities to persist. It is important to note that many of the new genomic tools may primarily benefit those who contribute to its design and development. AAs and other ethnically diverse populations remain underrepresented in clinical and research trials questioning the validity of genomic tools in AAs.

It is important to consider the limitations of this study. First, the records available for study, with the data of interest, were limited to 9,120 out of 62,838; but the data were still consistent with other studies. Another limitation is that the patient clinical treatment site is unknown and the site of the testing could not be identified. The lower utilization of ODX could be a reflection of the population served by the clinic or hospital center. However, this study was consistent with other studies in that AAs were less likely to utilize ODX although eligible.

To ameliorate the gaps in utilization and referral, it is recommended that physicians are educated on the benefits of precision medicine and the current tools available to personalize cancer treatment. This will mitigate the toxic effects of current treatment regimens and improve quality of life in survivors. We also recommend that researchers continue to develop tools that include underserved and ethnically diverse populations.

## Conflict of Interest

None declared.
